# Comparative Analysis of Plant Defense Activation by Four Biosurfactants: Mode of Action and Disease Control Potential

**DOI:** 10.3390/ijms26178313

**Published:** 2025-08-27

**Authors:** Yoshinao Aoki, Takayuki Asada, Masutoshi Nojiri, Shunji Suzuki

**Affiliations:** 1 Laboratory of Fruit Genetic Engineering, The Institute of Enology and Viticulture, University of Yamanashi, Kofu 400-0005, Japan; yaoki@yamanashi.ac.jp; 2 Agri-Bio Research Center, Kaneka Corporation, Iwata 438-0802, Japan

**Keywords:** downy mildew, grapevine, rhamnolipid, sophorolipid, spiculisporic acid, surfactin

## Abstract

Grapevine (*Vitis vinifera*) is highly susceptible to fungal diseases, particularly downy mildew caused by *Plasmopara viticola*. Environmental contamination and potential health risks to viticulturists have raised concerns about the long-term sustainability of chemical control. In this study, we evaluated the potential of four biosurfactants—surfactin, rhamnolipid, sophorolipid, and spiculisporic acid—as alternative agents to chemical fungicides for disease management in viticulture. Surfactin, rhamnolipid, and sophorolipid, but not spiculisporic acid, significantly reduced the severity of grape downy mildew and strawberry anthracnose and induced the expression of defense-related genes, such as β-1,3-glucanase and class IV chitinase, in grapevine and strawberry leaves, although each biosurfactant triggered distinct gene expression patterns. Utilizing salicylic acid (SA)- and jasmonate (JA)-insensitive mutants of *Arabidopsis thaliana*, we found that sophorolipid induced plant resistance through the canonical SA signaling pathway. In contrast, plant resistance induced by surfactin and rhamnolipid was independent of both the SA and JA signaling pathways. Notably, sophorolipid was the only biosurfactant that induced systemic acquired resistance in grapevine leaves through unknown signaling pathways, suppressing *P. viticola* infection at sites distant from the treatment area. These findings suggest that biosurfactants, particularly sophorolipids, are a promising eco-friendly alternative to conventional fungicides in viticulture.

## 1. Introduction

Grapevines are highly susceptible to various diseases and require extensive pest management strategies, including the application of chemical pesticides. Protecting grapevines against disease is a formidable task for viticulturists worldwide, and global warming is exacerbating the incidence of disease. In Hokkaido, a well-known wine-producing region located in the northernmost part of Japan, climate change has increased the incidence of grape downy mildew caused by *Plasmopara viticola*. Grape downy mildew had been uncommon in Hokkaido until recently, and its emergence has necessitated the use of chemical fungicides in vineyards [1]. High night temperatures promote the development of downy mildew disease in grapevines. Experiments have shown that elevated night temperatures enhanced *P. viticola* zoospore germination and hyphal growth on grapevine leaves, thereby promoting early infection by *P. viticola* [2]. In the context of global warming, viticulturists should closely monitor prolonged periods of elevated temperatures and proactively adjust their pest management strategies to prevent phytopathogen infections in grapevines.

A conventional and widely adopted strategy for mitigating damage caused by phytopathogen infections in grapevines is the application of chemical fungicides. Although effective in controlling several diseases, the long-term and repeated use of agrochemicals has raised significant concerns regarding environmental contamination and potential health risks to viticulturists and consumers. In response to these growing concerns, there has been increasing interest in developing and implementing alternative, sustainable disease management practices in viticulture. These approaches include the use of biocontrol agents [3] and/or natural bioactive compounds extracted from plants or microorganisms [4]. Environmentally friendly formulations exert antagonistic effects on phytopathogen growth [5,6] or induce systemic acquired resistance in plants, thereby inhibiting phytopathogen infection [7]. Some natural bioactive compounds possess bifunctional properties, namely, direct antagonistic activity and the ability to induce plant resistance. For example, the lipopeptide antimicrobial iturin A induced the expression of pathogenesis-related (PR) proteins in strawberry leaves [8].

Among biological alternatives, biosurfactants represent a particularly promising research area due to their dual functionality as both antimicrobial agents and plant defense inducers. Biosurfactants are natural surfactants produced by microorganisms. Biosurfactants are amphipathic molecules that contain both hydrophilic and hydrophobic moieties. They are well known for their distinctive physicochemical properties, such as the ability to self-assemble, reduce surface and interfacial tension, emulsify immiscible liquids, and adsorb to various surfaces. These characteristics make biosurfactants highly versatile and valuable in a wide range of applications, including environmental cleaning, sustainable agriculture, pharmaceuticals, and enhanced oil recovery [9]. One of the major advantages of biosurfactants is their high biodegradability and low toxicity, which contribute to their environmentally friendly profile. Furthermore, many biosurfactants exhibit biological activities, including direct antimicrobial and antiviral properties [10], making them promising candidates for applications where human health safety and ecological balance are a priority.

The objective of this study was to evaluate the potential for plant resistance induction by four biosurfactants—surfactin, rhamnolipid, sophorolipid, and spiculisporic acid—for future use in viticulture. These biosurfactants represent structurally diverse compounds derived from different microbial sources. Surfactin is a cyclic lipopeptide-type biosurfactant primarily produced by *Bacillus*. It is characterized by a unique structure in which seven amino acids are linked to form a cyclic ring [11]. Rhamnolipid and sophorolipid are glycolipid-type biosurfactants produced by *Pseudomonas* [12] and non-pathogenic yeasts [13], respectively. Spiculisporic acid is a fatty acid-type biosurfactant originally isolated from *Penicillium spiculisporum*, containing two carboxyl groups and a lactone ring [14]. Its unique structural properties allow for the synthesis of various spiculisporic acid derivatives with a wide range of surfactant activities [15]. All these biosurfactants possess antimicrobial activity; however, surfactin [16] and rhamnolipid [17] have been demonstrated to induce disease resistance in plants. In this study, we demonstrated that surfactin, rhamnolipid, and sophorolipid, but not spiculisporic acid, induced disease resistance in grapevine. Notably, sophorolipid specifically elicited systemic acquired resistance in grapevine leaves. These findings suggest that biosurfactants, particularly sophorolipids, are promising agents for sustainable disease management in viticulture.

## 2. Results

### 2.1. Effect of Surfactin, Rhamnolipid, Sophorolipid, and Spiculisporic Acid on Grape Downy Mildew in Grapevine Leaf Disks

An in vivo bioassay was performed using grapevine leaf disks to evaluate the effects of biosurfactants against downy mildew, where biosurfactant solutions were applied to leaf disks followed by *P. viticola* inoculation. Disease severity was assessed 7 days post-inoculation using a 0–5 scale based on white symptom coverage on the leaf disks, as described in Materials and Methods.

**Figure 1:**
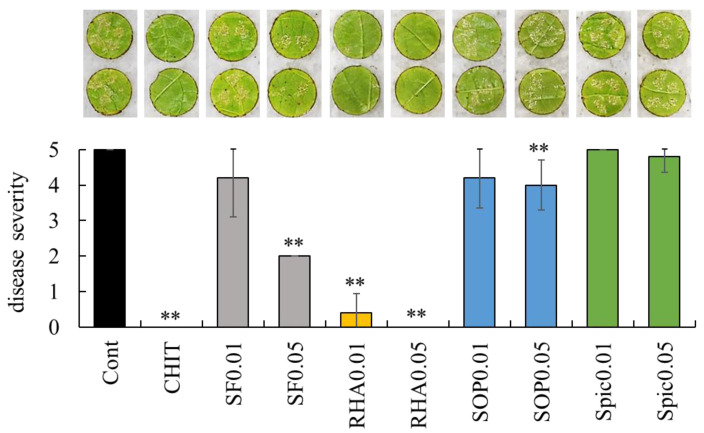
Effect of biosurfactants on grape downy mildew. Representative images of grape downy mildew symptoms on leaf disks treated with sterilized water, chitosan, and biosurfactants are presented above the graph. Disease severity was evaluated as described in Materials and Methods. Bars indicate means ± standard deviations from five replicate leaf disks per treatment. ** *p* < 0.01 compared with control (sterilized water). Cont, treated with sterilized water (control). CHIT, treated with 1000 mg/mL chitosan. SF0.01 and SF0.05, treated with 0.01 and 0.05 mg/mL surfactin, respectively. RHA0.01 and RHA0.05, treated with 0.01 and 0.05 mg/mL rhamnolipid, respectively. SOP0.01 and SOP0.05, treated with 0.01 and 0.05 mg/mL sophorolipid, respectively. Spic0.01 and Spic0.05, treated with 0.01 and 0.05 mg/mL spiculisporic acid, respectively.

A large number of *P. viticola* white symptoms were observed on the leaf disks treated with sterilized water. In contrast, symptom development was markedly suppressed on the leaf disks treated with chitosan, which, as reported previously [18], reduced downy mildew severity on grapevine leaves (Figure 1). Disease severity was significantly reduced by surfactin (0.05 mg/mL), rhamnolipid (0.01 and 0.05 mg/mL), and sophorolipid (0.05 mg/mL) compared with control (sterilized water). Notably, rhamnolipid at 0.05 mg/mL completely suppressed the development of *P. viticola* symptoms. These inhibitory effects of the biosurfactants on *P. viticola* infection appeared to be dose-dependent. A large number of white symptoms were visible on the disk treated with spiculisporic acid.

### 2.2. Effect of Surfactin, Rhamnolipid, Sophorolipid, and Spiculisporic Acid on Strawberry Anthracnose in Strawberry Leaflets

The strawberry—*C. gloeosporioides* pathosystem was used as a surrogate model for grape ripe rot caused by *Colletotrichum* species. An in vivo bioassay using strawberry leaflets was performed to evaluate the effects of biosurfactants against *C. gloeosporioides* infection, in which biosurfactants were applied to detached leaflets followed by *C. gloeosporioides* inoculation. Disease severity was assessed 10 days post-inoculation using a 0–5 scale based on lesion diameter, as described in Materials and Methods.

**Figure 2:**
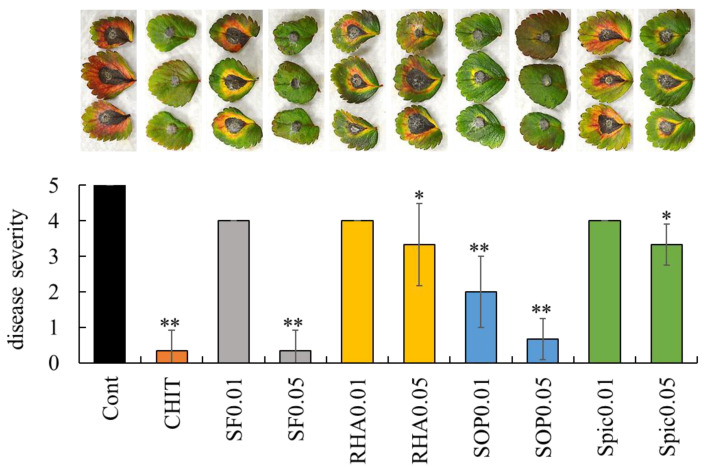
Effect of biosurfactants on strawberry anthracnose. Representative images of anthracnose lesions on strawberry leaflets treated with sterilized water, chitosan, and biosurfactants are presented above the graph. Disease severity was evaluated as described in Materials and Methods. Bars indicate means ± standard deviations from three replicate leaflets per treatment. * *p* < 0.05 compared with control (sterilized water). ** *p* < 0.01 compared with control (sterilized water). Cont, treated with sterilized water (control). CHIT, treated with 1000 mg/mL chitosan. SF0.01 and SF0.05, treated with 0.01 and 0.05 mg/mL surfactin, respectively. RHA0.01 and RHA0.05, treated with 0.01 and 0.05 mg/mL rhamnolipid, respectively. SOP0.01 and SOP0.05, treated with 0.01 and 0.05 mg/mL sophorolipid, respectively. Spic0.01 and Spic0.05, treated with 0.01 and 0.05 mg/mL spiculisporic acid, respectively.

Large lesions caused by *C. gloeosporioides* were clearly observed on the leaflets treated with sterilized water, whereas lesion development was suppressed on the leaflet treated with chitosan (Figure 2). Surfactin (0.05 mg/mL), rhamnolipid (0.05 mg/mL), sophorolipid (0.01 and 0.05 mg/mL), and spiculisporic acid (0.05 mg/mL) significantly reduced the disease severity compared with control (sterilized water). Notably, surfactin at 0.05 mg/mL suppressed lesion development by *C. gloeosporioides* to a level of disease severity comparable to that observed with chitosan treatment.

### 2.3. Effect of Surfactin, Rhamnolipid, Sophorolipid, and Spiculisporic Acid on Gene Expression of PR Proteins in Grapevine Leaves and Strawberry Leaflets

**Figure 3:**
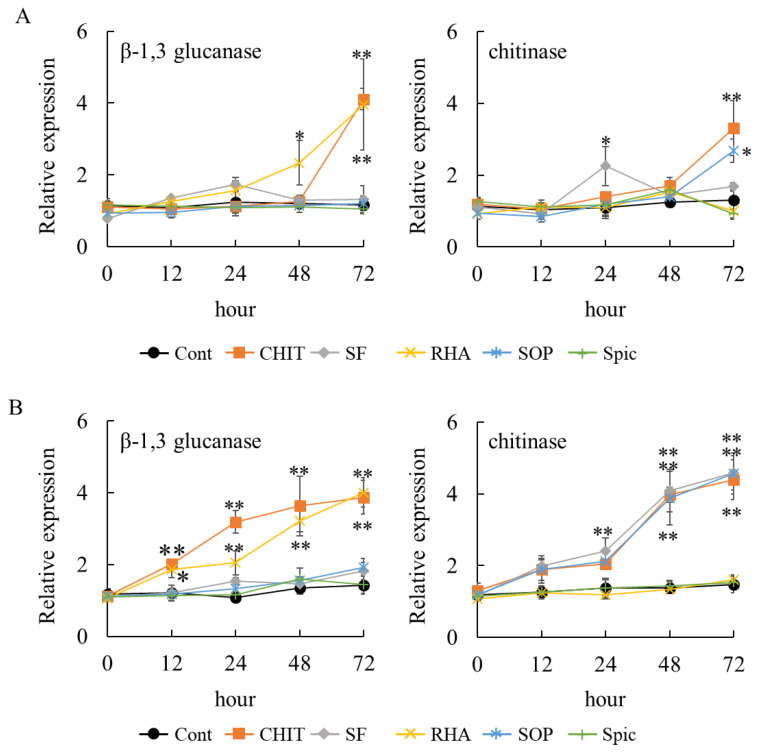
Effect of biosurfactants on the gene expression of β-1,3-glucanase and class IV chitinase. To evaluate the potential of each biosurfactant for inducing plant resistance, grapevine leaf disks (**A**) and strawberry leaflets (**B**) were treated with biosurfactants, chitosan, or sterilized water and subsequently analyzed by real-time RT-PCR. Data were calculated as gene expression relative to β-actin expression. Bars indicate means ± standard deviations from three replicates per treatment. * *p* < 0.05 compared with control (sterilized water). ** *p* < 0.01 compared with control (sterilized water). Cont, treated with sterilized water (control). CHIT, treated with 1000 mg/mL chitosan. SF, treated with 0.05 mg/mL surfactin. RHA, treated with 0.05 mg/mL rhamnolipid. SOP, treated with 0.05 mg/mL sophorolipid. Spic, treated with 0.05 mg/mL spiculisporic acid.

β-1,3-glucanase and chitinase were selected as markers of plant defense activation because they are well-characterized PR proteins that are rapidly induced during plant defense responses [19]. In grapevines, both enzymes serve as reliable biochemical indicators of defense activation and have been widely used to assess resistance induction by various elicitors [20,21]. To evaluate the potential of surfactin, rhamnolipid, sophorolipid, and spiculisporic acid to induce plant resistance, these biosurfactants were applied to grapevine leaf disks and strawberry leaflets. In grapevine leaf disks, chitosan induced the gene expression of both β-1,3-glucanase and class IV chitinase at 72 h post-treatment (Figure 3A). Among the four biosurfactants tested, only rhamnolipid induced the gene expression of β-1,3-glucanase at 48 h post-treatment, and its expression level further increased at 72 h. Surfactin induced the gene expression of class IV chitinase at 24 h post-treatment, whereas sophorolipid induced its expression at 72 h post-treatment. Spiculisporic acid failed to induce the gene expression of β-1,3-glucanase or class IV chitinase.

In strawberry leaflets, chitosan induced the expression of both β-1,3-glucanase and class IV chitinase genes at 12 h and 48 h post-treatment, respectively, and the expression levels further increased at 72 h (Figure 3B). Similar to grapevine, among the four biosurfactants tested, only rhamnolipid induced the gene expression of β-1,3-glucanase at 12 h post-treatment; its expression level further increased at 72 h. Likewise, surfactin and sophorolipid induced the gene expression of class IV chitinase at 24 h and 48 h post-treatment, respectively, with the expression level further increased at 72 h. Spiculisporic acid did not induce the gene expression of β-1,3-glucanase or class IV chitinase.

These results suggest that surfactin, rhamnolipid, and sophorolipid, but not spiculisporic acid, have the potential to induce plant resistance. However, each biosurfactant exhibited a distinct gene expression pattern.

### 2.4. Assessment of Biosurfactant-Induced Resistance via SA- and/or JA-Dependent Defense Pathways

To determine whether plant resistance induced by surfactin, rhamnolipid, and sophorolipid occurs via SA- and/or JA-dependent defense pathways, rosette leaves of SA-insensitive or JA-insensitive *Arabidopsis* mutants were treated with these biosurfactants, and the expression of *PR1* [22] and *PDF1.2* [23] in rosette leaves was analyzed as markers of SA- and JA-dependent defense pathway, respectively.

**Figure 4:**
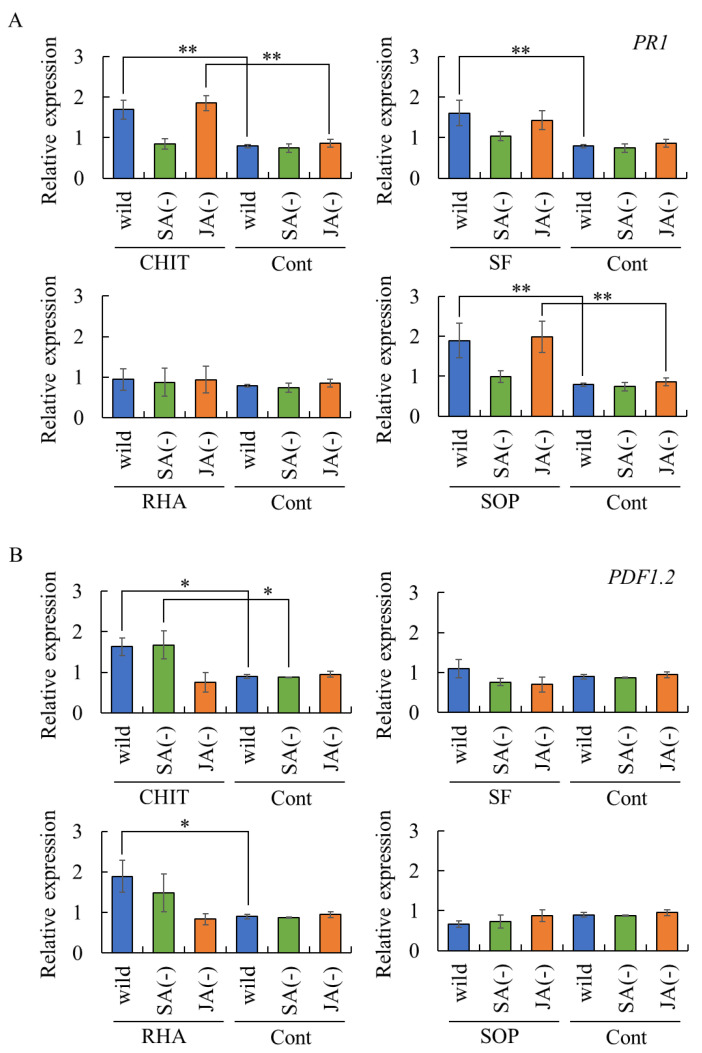
Expression of *PR1* and *PDF1.2* in *Arabidopsis* rosette leaves treated with biosurfactants. Rosette leaves of *A. thaliana* Col-0 (wild type), the *npr1-5* mutant (SA-insensitive), and the *atmyc2* mutant (JA-insensitive) were treated with surfactin, rhamnolipid, sophorolipid, chitosan, or sterilized water. After 72 h post-incubation, the treated rosette leaves were analyzed by real-time RT-PCR to evaluate the transcript levels of *PR1* (**A**) and *PDF1.2* (**B**). Data were calculated as gene expression relative to β-actin expression. Bars indicate means ± standard deviations from three replicates per treatment. * *p* < 0.05 compared with control (sterilized water). ** *p* < 0.01 compared with control (sterilized water). Wild, wild type Col-0. SA(−), SA-insensitive mutant *npr1-5*. JA(−), JA-insensitive mutant *atmyc2.* Cont, treated with sterilized water (control). CHIT, treated with 1000 mg/mL chitosan. SF, treated with 0.05 mg/mL surfactin. RHA, treated with 0.05 mg/mL rhamnolipid. SOP, treated with 0.05 mg/mL sophorolipid.

Chitosan treatment induced the expression of *PR1* and *PDF1.2* in rosette leaves, reflecting the activation of the SA- and JA-dependent defense pathways, respectively (Figure 4). These results confirm that the experimental system is appropriate for evaluating SA- and JA-mediated defense responses.

Surfactin induced the expression of *PR1*, but not *PDF1.2*, in rosette leaves of wild-type *Arabidopsis* plants (Figure 4). However, *PR1* expression was not upregulated in surfactin-treated rosette leaves of SA- or JA-insensitive mutants, suggesting that surfactin induced *PR1* expression independent of the SA- and JA-dependent defense pathways. Rhamnolipid induced the expression of *PDF1.2*, but not *PR1*, in rosette leaves of wild-type *Arabidopsis* plants. However, *PDF1.2* expression was not upregulated in rhamnolipid-treated rosette leaves of SA- or JA-insensitive mutants, suggesting that rhamnolipid does not induce *PDF1.2* expression through the SA- and JA-dependent defense pathways.

Sophorolipid did not induce *PDF1.2* expression in rosette leaves of either *Arabidopsis* genotype (Figure 4B). In contrast, *PR1* expression was observed in rosette leaves of sophorolipid-treated wild-type plants and the JA-insensitive mutant, but not the SA-insensitive mutant (Figure 4A), indicating that sophorolipid activates the SA-dependent defense pathway.

Taken together, these findings suggest that biosurfactants can activate plant immune responses, although some, such as surfactin and rhamnolipid, may act through alternative, yet unknown signaling pathways.

### 2.5. Assessment of Systemic Acquired Resistance Triggered by Biosurfactants

**Figure 5:**
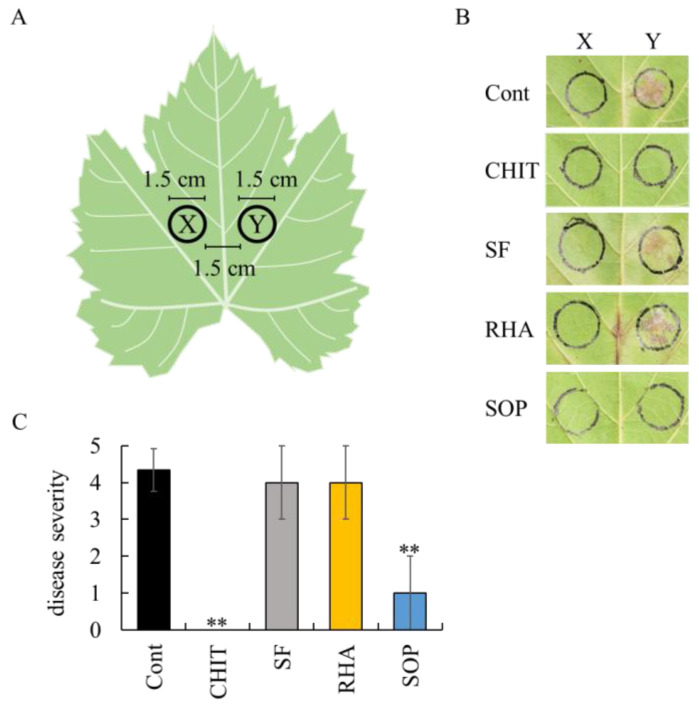
Effect of biosurfactants on grape downy mildew at grapevine leaf sites distal to the treated area. (**A**) Schematic representation of the experiment. Biosurfactants were applied to one of two marked areas (1.5 cm diameter, X in the figure) on the abaxial surface of grapevine leaves. *P. viticola* zoosporangia were applied to the opposite, untreated area (1.5 cm diameter, Y in the figure), positioned 1.5 cm apart from the treated area (X). (**B**) Representative images of grape downy mildew symptoms on treated area (X) and untreated area (Y). (**C**) Disease severity on untreated area (Y) was evaluated as described in Materials and Methods. Bars indicate means ± standard deviations from three replicate leaves per treatment. ** *p* < 0.01 compared with control (sterilized water). Cont, treated with sterilized water (control). CHIT, treated with 1000 mg/mL chitosan. SF, treated with 0.05 mg/mL surfactin. RHA, treated with 0.05 mg/mL rhamnolipid. SOP, treated with 0.05 mg/mL sophorolipid.

To determine whether biosurfactants can induce systemic acquired resistance, their ability to suppress *P. viticola* infection was assessed at grapevine leaf sites distant from the biosurfactant application area, as described in Materials and Methods and illustrated in Figure 5.

A large number of *P. viticola* white symptoms were observed at the site distant from the sterilized water application site. In contrast, chitosan markedly suppressed such symptoms at the site distant from the application site (Figure 5B). These findings reflect the activation of systemic acquired resistance by chitosan, thereby validating the experimental system. Surfactin and rhamnolipid did not induce systemic acquired resistance in grapevine leaves, as *P. viticola* white symptoms were detected at sites distant from the application site (Figure 5B). Among the biosurfactants tested, only sophorolipid effectively suppressed *P. viticola* infection at the site distant from the application site. This resulted in a significant reduction in disease severity (Figure 5C). Since the key characteristic of systemic acquired resistance is its ability to extend protection beyond the site of initial treatment, reaching untreated, distant plant tissues through systemic signaling pathways [24], this result suggest that sophorolipid is capable of inducing systemic acquired resistance in grapevine leaves.

**Figure 6:**
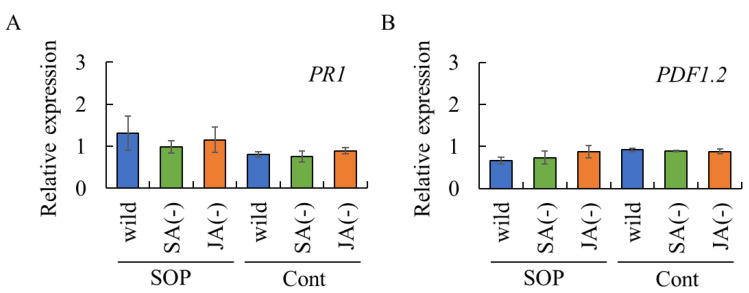
Expression of *PR1* and *PDF1.2* in *Arabidopsis* rosette leaves untreated with sophorolipid. Rosette leaves of *A. thaliana* Col-0 (wild type), the *npr1-5* mutant (SA-insensitive), and *atmyc2* mutant (JA-insensitive) were treated with sophorolipid or sterilized water. After 72 h post-incubation, the untreated rosette leaves were analyzed by real-time RT-PCR to evaluate the transcript levels of *PR1* (**A**) and *PDF1.2* (**B**). Data were calculated as gene expression relative to β-actin expression. Bars indicate means ± standard deviations from three replicate leaves per treatment. Cont, treated with sterilized water (control). SOP, treated with 0.05 mg/mL sophorolipid.

Next, to determine whether the systemic acquired resistance induced by sophorolipid occurs via SA- and/or JA-dependent defense pathways, rosette leaves of SA-insensitive or JA-insensitive *Arabidopsis* mutants were treated with sophorolipid, and untreated leaves were analyzed for *PR1* and *PDF1.2* expression. Neither gene was induced in untreated leaves, indicating that the SA- and JA-dependent defense pathways are not involved in the systemic acquired resistance induced by sophorolipid (Figure 6).

## 3. Discussion

**Figure 7:**
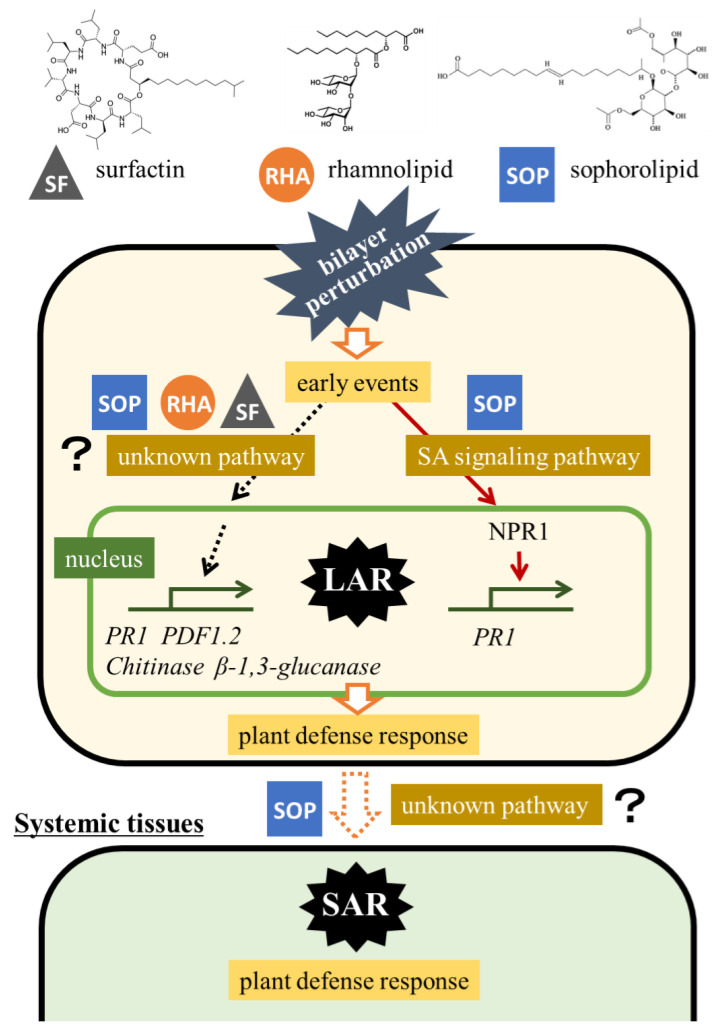
Predicted defense pathways activated in plants exposed to biosurfactants. LAR, local acquired resistance. SAR, systemic acquired resistance. NPR1, nonexpressor of pathogenesis-related genes 1. SF, surfactin. RHA, rhamnolipid. SOP, sophorolipid.

In this study, we demonstrated that certain biosurfactants, particularly surfactin, rhamnolipid, and sophorolipid, are capable of inducing disease resistance in grapevine. Only sophorolipid induced systemic acquired resistance in grapevine, independent of the SA and JA signaling pathways. Figure 7 shows the predicted signaling pathways involved in plant resistance triggered by the biosurfactants. Biosurfactants interact with the cell membrane lipid bilayer, compromising membrane integrity. This leads to the exposure of membrane proteins on the cell surface, which further affects cell function and stability [25]. These interactions guide the cells toward early events that trigger the plant defense response at the local site exposed to biosurfactants. When the SA signaling pathway is activated, nonexpressor of pathogenesis-related genes 1 (NPR1) is transported to the nucleus [26], where it upregulates the expression of defense-related genes, such as PR1. Among the biosurfactants tested in this study, only sophorolipid activated this pathway.

Sophorolipid was first discovered as a substance produced by the yeast *Candida magnoliae* [27]. Today, sophorolipids are applied in toiletries, cosmetics, medicine, and agriculture owing to their surface activity, emulsifying ability, and antimicrobial properties [28]. The antimicrobial properties of sophorolipids have been demonstrated in vitro and in vivo. In vitro, they inhibit hyphal growth in *Pythium ultimum*, *Rhizoctonia solani*, *Sclerotium rolfsii*, and *Botrytis cinerea* through cellular modifications. In vivo, the application of sophorolipids to tomato leaves and fruits reduces necrosis caused by *B. cinerea* [29]. The antimicrobial activity of sophorolipids is thought to occur through the destabilization of fungal cell membrane, possibly leading to cytoplasmic extrusion and death [30]. *P. viticola* is an oomycete like *P. ultimum*, and *C. gloeosporioides* is a fungus. Although we found that sophorolipid induced both local and systemic resistance in grapevines, the reduction in grape downy mildew and strawberry anthracnose disease severity by sophorolipid could be attributed to its antimicrobial properties. Our findings suggest that sophorolipid plays a dual role in disease control: it enhances plant resistance and exerts direct antimicrobial effects against phytopathogens. Future field trials with sophorolipid in vineyards may demonstrate its effectiveness for controlling a broad range of fungal diseases in viticulture.

Surfactin and rhamnolipid also reduced grape downy mildew and strawberry anthracnose disease severities. They also induced PR protein gene expression in grapevine, strawberry, and *Arabidopsis* plants. However, gene transcription did not occur through the SA or JA signaling pathway. Previous studies have demonstrated that surfactin [16] and rhamnolipid [17] induce disease resistance in plants. Surfactin has been shown to induce resistance to *Fusarium* wilt in watermelon through the activation of defense-related genes involved in JA and SA signaling [31]. The SA signaling pathway also plays a vital role in rhamnolipid-induced resistance in *Arabidopsis* plants [17]. These findings contradict the results of this study, which used SA- and JA-insensitive mutants treated with surfactin and rhamnolipid. This discrepancy may not be solely due to treatment conditions but could also result from differences in the type or purity of the surfactin and rhamnolipid used, or the timing of gene expression analysis. In this study, the earliest time point examined for gene expression analysis was 12 h post-treatment. However, some defense-related genes associated with systemic acquired resistance or local acquired resistance can be activated within just a few hours after treatment. Therefore, responses occurring at earlier stages may have been overlooked in our analysis. Moreover, complex crosstalk between the SA and JA signaling pathways, as well as interactions with other pathways, such as the ethylene signaling pathway, may also contribute to the observed differences. Even while collecting research data on plant resistance induced by surfactin and rhamnolipid, their antimicrobial properties are thought to contribute to protection against fungi and oomycetes by causing structural damage to spores and fungal hyphae [32,33]. Together with previous research, our findings underscore the potential of surfactin and rhamnolipid as a new class of disease control agents, combining the ability to elicit plant immune responses with direct antimicrobial effects against pathogens in viticulture.

In viticulture, downy mildew remains a significant challenge, partly due to its increasing resistance to fungicides. This is because *P. viticola* is considered a high-risk phytopathogen that readily acquires resistance to chemical fungicides [34]. Given their dual role in inducing plant defense responses, as demonstrated in this study, and directly inhibiting phytopathogens [30,32,33], surfactin, rhamnolipid, and sophorolipid have emerged as promising alternatives to synthetic fungicides for disease management in viticulture. For practical application, it will be essential to develop tailored methods for downy mildew control, including optimization of timing, dosage, and formulation. Moreover, exploring the combined use of different biosurfactants may further enhance disease suppression by activating multiple signaling pathways involved in plant resistance. These efforts are expected to pave the way for a new generation of sustainable disease management strategies in viticulture.

## 4. Materials and Methods

### 4.1. Chemicals

Surfactin sodium salt (97% purity) and sophorolipid (40% purity) were obtained from Kaneka (Tokyo, Japan). Rhamnolipid (90% purity) and spiculisporic acid (98% purity) were purchased from Sigma-Aldrich (Steinheim, Germany) and Tokyo Chemical Industry (Tokyo, Japan), respectively. Chitosan 100 was purchased from FUJIFILM Wako (Osaka, Japan) as an elicitor of disease resistance in grapevine [18].

The chitosan and biosurfactant concentrations tested were selected based on previous studies that demonstrated their effectiveness in inducing plant defense responses [16,17,18].

### 4.2. Plant Materials

Grapevine seedlings (*Vitis* sp. cv. Koshu) were grown on their own roots in pots at 27 °C under light irradiation (11.8 Wm^−2^/16 h/d). Sixty-day-old seedlings were used in the following experiments.

*Fragaria* × *ananassa* cv. Nyoho seedlings were cultivated in a soil mixture consisting of 55% peat moss, 10% perlite, 5% vermiculite, and 30% decomposed granite soil, at 25 °C under light irradiation (11.8 Wm^−2^/16 h/d). Sixty-day-old seedlings were used in the following experiments.

Seeds of *Arabidopsis thaliana* wild type (Col-0), the salicylic acid (SA)-insensitive mutant *npr1-5* (CS3724) [26], and the jasmonate (JA)-insensitive mutant *atmyc2* (SALK_039235) [35] were obtained from The Arabidopsis Information Resource. Seeds were sown on rockwool blocks and incubated at 22 °C under light irradiation (11.8 W m^−2^/16 h/d). Thirty-day-old seedlings were used in the following experiments.

### 4.3. In Vivo Bioassay for Downy Mildew Disease Severity in Grapevine Leaf Disks

An in vivo bioassay using grapevine leaf disks was performed to evaluate whether biosurfactants suppress grape downy mildew, following a previously reported method [36]. Briefly, the third to fifth leaves from the shoot apex of grapevine seedlings were collected. Leaf disks were excised from the leaves using a cork borer (13 mm diameter) and placed abaxial side up on moistened filter paper in Petri dishes (140 mm × 100 mm). Biosurfactant solutions were prepared at concentrations of 0.01 and 0.05 mg/mL. Ten µL of each solution was applied to four locations on the abaxial surface of each leaf disk. Sterilized water and chitosan (1000 mg/mL) were used as controls. The treated leaf disks were air-dried in a flow cabinet at room temperature for 6 h.

*Plasmopara viticola* (Berk. & M.A. Curtis) Berl. & De Toni was maintained on Koshu leaves in an incubator at 27 °C under light irradiation (11.8 W m^−2^/16 h/d). Fresh zoosporangia of *P. viticola* were collected by washing symptomatic leaves with sterilized water. Ten µL of the zoosporangia suspension (1 × 10^4^ zoosporangia/mL) was dropped on the same four locations previously treated with biosurfactant solutions on each leaf disk. Petri dishes containing the treated leaf disks were placed in a plastic box with moistened paper towels and incubated at 22 °C under the same light conditions (11.8 W m^−2^/16 h/d).

Downy mildew symptoms on each disk were evaluated 7 d after *P. viticola* inoculation. Disease severity was scored on the basis of the extent of white symptoms on each disk, as follows: 0, no symptoms; 1, white symptoms covering up to 1/6 of the disk; 2, up to 1/3 of the disk; 3, up to 1/2 of the disk; 4, up to 2/3 of the disk; 5, more than 2/3 of the disk. The mean disease severity score was calculated from five replicate leaf disks per treatment in each experiment.

### 4.4. In Vivo Bioassay for Anthracnose Disease Severity in Strawberry Leaves

Strawberry leaves were used as the alternative host of *Colletotrichum gloeosporioides*, causing grape ripe rot, because it was difficult to evaluate the symptoms of *C. gloeosporioides* on grapevine leaves. An in vivo bioassay using strawberry leaves was performed to evaluate whether biosurfactants suppress strawberry anthracnose caused by *C. gloeosporioides*, following a previously described method [37].

Leaflets were detached from strawberry seedlings, and the detached leaflets were placed adaxial side up on moistened filter paper in Petri dishes (140 mm × 100 mm). Two hundred µL of each biosurfactant solution (0.01 and 0.05 mg/mL) was applied to the center of each leaflet. Sterilized water and chitosan (1000 mg/mL) were used as controls. The treated leaflets were air-dried in a flow cabinet at room temperature for 6 h. The center of each leaflet was punctured with a sterile needle of 1 mm diameter.

A *C. gloeosporioides* laboratory strain isolated from the experimental vineyard of The Institute of Enology and Viticulture, University of Yamanashi, was used in this study. The fungus was cultured on a potato dextrose agar (PDA, Becton and Dickinson, Andover, MA, USA) plate at 25 °C for 10 d. Agar plugs (6 mm in diameter) containing actively growing *C. gloeosporioides* were prepared using a cork borer, and a single plug was placed upside down onto a wound of each leaflet. Petri dishes containing the treated leaflets were placed in a plastic box with moistened paper towels and incubated at 25 °C under light conditions (11.8 W m^−2^/16 h/d). On day 10 post-incubation, the diameter of the lesion on each leaflet was measured to assess anthracnose disease severity.

Disease severity was scored as follows: 0, no symptoms; 1, rot limited to the area beneath the agar plug; 2, rot approximately twice the diameter of the plug; 3, three times the diameter of the plug; 4, four times the diameter of the plug; 5, more than five times the diameter of the plug. The mean disease severity score was calculated from three replicate leaflets per treatment in each experiment.

### 4.5. Treatment of Arabidopsis Rosette Leaves with Each Biosurfactant

Rosette leaves of 30-day-old *Arabidopsis* seedlings growing in pots were used. Forty μL of 0.05 mg/mL of each biosurfactant, 1000 mg/mL chitosan, or sterilized water were carefully placed on the adaxial surface of individual rosette leaves using a micropipette, and air-dried at room temperature. The seedlings were incubated at 22 °C in an incubator (11.8 Wm^−2^/16 h/d). After 72 h incubation, the treated and untreated rosette leaves were subjected to real-time RT-PCR.

### 4.6. Total RNA Isolation

Grapevine leaf disks, strawberry leaflets, and *Arabidopsis* rosette leaves were treated with 0.05 mg/mL of each biosurfactant, 1000 mg/mL chitosan, or sterilized water, as described above. After incubation for the indicated period at 25 °C under light conditions (11.8 W m^−2^/16 h/day), the treated samples were frozen in liquid nitrogen and ground with a pestle. Total RNA was isolated from each pulverized sample using a Fruit-mate for RNA Purification (Takara, Otsu, Japan) and an RNeasy Plant Mini Kit (Qiagen, Hilden, Germany), with an automated QIAcube (Qiagen) following the manufacturer’s instructions.

### 4.7. cDNA Synthesis

First-strand cDNA was synthesized from total RNA using a PrimeScript RT Reagent Kit with gDNA Eraser (Takara, Shiga, Japan) following the manufacturer’s instructions.

### 4.8. Real-Time RT-PCR

Real-time RT-PCR was performed with TB Green Premix EX Taq II (Tli RNaseH Plus) (Takara) with a Thermal Cycler Dice Real-Time System TP980 (Takara) in accordance with the manufacturer’s instructions. PCR amplification was performed for 40 cycles at 95 °C for 5 s and at 60 °C for 45 s, and for one cycle at 95 °C for 15 s, at 60 °C for 30 s, and at 95 °C for 15 s after an initial denaturation at 95 °C for 30 s. The nucleotide sequences of the primers used for real-time RT-PCR were as follows: *V. vinifera* class IV chitinase primers (5′-CAATCGGGTCCTTGTGATTC-3′ and 5′-CAAGGCACTGAGAAACGCT-3′, GenBank accession no. U97522), *V. vinifera* β-1,3-glucanase primers (5′-GAATCTGTTCGATGCCATGC-3′ and 5′-GCATTATCAACCGTAGTCCC-3′, GenBank accession no. DQ267748), *V. vinifera* β-actin primers (5′-CAAGAGCTGGAAACTGCAAAGA-3′ and 5′-AATGAGAGATGGCTGGAAGAGG-3′, GenBank accession no. AF369524), *Fragaria* × *ananassa* class IV chitinase primers (5′-GATGCCCTCGGCTCTTACAC-3′ and 5′-CGCAGTAGGTGGCCTTTTCT-3′, GenBank accession no. JN415653), *Fragaria* × *ananassa* β-1,3-glucanase primers (5′-ATCTGCGGGTGTCTGCTATG-3′ and 5′-TAAGGGCTTCCAAGGTTGCT-3′, GenBank accession no. AY170376), *Fragaria* × *ananassa* actin primers (5′-GTGCGACAATGGAACTGGAA-3′ and 5′-CTTTCTGCCCCATACCAACC-3′, GenBank accession no. JN616288), *A. thaliana* PR1 primers (5′-CCTGGGGTAGCGGTGACTT-3′ and 5′-CGTGTTCGCAGCGTAGTTGT-3′, GenBank accession no. NM_127025), *A. thaliana* PDF1.2 primers (5′-TCACCCTTATCTTCGCTGCTC-3′ and 5′-ACCATGTCCCACTTGGCTTC-3′, GenBank accession no. AY063779), and *A. thaliana* actin primers (5′-GCCGACAGAATGAGCAAAGAG-3′ and 5′-AGGTACTGAGGGAGGCCAAGA-3′, GenBank accession no. NM_179953).

Dissociation curves were analyzed to verify the specificity of the amplification reaction. Gene expression levels were quantified using the standard curve method with Thermal Cycler Dice Real Time System Software (ver. 5.11, Takara), based on the number of amplification cycles required to reach a fixed threshold (Ct value). Data are expressed as values relative to each actin and presented as means ± standard deviations.

### 4.9. Possible Systemic Acquired Resistance in Grapevine Leaves

The fifth to seventh leaves from the shoot apex of grapevine seedlings were collected. Two circles, each with a diameter of 1.5 cm, were drawn on the abaxial surface of each leaf using a marker pen. The circles were positioned on either side of the central vein, with a 1.5 cm gap between them. Forty µL each of biosurfactant (0.05 mg/mL), chitosan (1000 mg/mL), or sterilized water was dropped onto one of the marked circles on each leaf. After air-drying the leaves at room temperature for 6 h, 40 µL of *P. viticola* zoosporangia suspension (1 × 10^4^ zoosporangia/mL) was dropped onto the opposite circles (not treated with biosurfactants). The treated leaves were placed abaxial side up on moistened paper towels in a plastic box and incubated at 25 °C in the dark, followed by incubation at 25 °C under light conditions (11.8 W m^−2^/16 h/d).

Downy mildew symptoms on each circle were evaluated 10 d after *P. viticola* inoculation. Disease severity was scored on the basis of the extent of white symptoms within each circle, as follows: 0, no symptoms; 1, white symptoms covering up to 1/6 of the circle; 2, up to 1/3 of the circle; 3, up to 1/2 of the circle; 4, up to 2/3 of the circle; 5, more than 2/3 of the circle. The mean disease severity score was calculated from three replicate leaves per treatment in each experiment.

### 4.10. Statistical Analysis

Data are presented as means ± standard deviations of biological replicates. Statistical analysis was performed using Dunnett’s test in Excel statistics software 2012.

## Data Availability

Data is contained within the article.

## Abbreviations

The following abbreviations are used in this manuscript:

JA	Jasmonate
PR protein	Pathogenesis-related protein
SA	Salicylic acid

## References

[1] Aoki Y., Kasai Y., Ikehara S., Sasada T., Suzuki S. (2019). Monitoring of QoI fungicide resistance in *Plasmopara viticola* populations in Hokkaido. J. ASEV Jpn..

[2] Aoki Y., Usujima A., Suzuki S. (2021). High night temperature promotes downy mildew in grapevine via attenuating plant defense response and enhancing early *Plasmopara viticola* infection. Plant Prot. Sci..

[3] Karačić V., Miljaković D., Marinković J., Ignjatov M., Milošević D., Tamindžić G., Ivanović M. (2024). *Bacillus* species: Excellent biocontrol agents against tomato diseases. Microorganisms.

[4] Soković M.D., Glamočlija J.M., Ćirić A.D., Nita M. (2013). Natural products from plants and fungi as fungicides. Fungicides-Showcases of Integrated Plant Disease Management from Around the World.

[5] Ranade Y., Pathak P., Chandrashekar M., Saha S. (2023). Biological control of *Colletotrichum gloeosporioides* (Penz.) Penz. & Sacc. by epiphytic bacteria isolated from *Vitis vinifera* (cv Thompson Seedless) grape berry. Biocontrol Sci. Technol..

[6] Sun Z.-B., Song H.-J., Liu Y.-Q., Ren Q., Wang Q.-Y., Li X.-F., Pan H.-X., Huang X.-Q. (2024). The potential of microorganisms for the control of grape downy mildew—A review. J. Fungi.

[7] Ishiai S., Kondo H., Hattori T., Mikami M., Aoki Y., Enoki S., Suzuki S. (2016). Hordenine is responsible for plant defense response through jasmonate-dependent defense pathway. Physiol. Mol. Plant Pathol..

[8] Yamamoto S., Shiraishi S., Suzuki S. (2015). Are cyclic lipopeptides produced by *Bacillus amyloliquefaciens* S13-3 responsible for the plant defense response in strawberry against *Colletotrichum gloeosporioides*?. Lett. Appl. Microbiol..

[9] Romero Vega G., Gallo Stampino P. (2025). Bio-based surfactants and biosurfactants: An overview and main characteristics. Molecules.

[10] Puyol McKenna P., Naughton P.J., Dooley J.S.G., Ternan N.G., Lemoine P., Banat I.M. (2024). Microbial biosurfactants: Antimicrobial activity and potential biomedical and therapeutic exploits. Pharmaceuticals.

[11] Peypoux F., Bonmatin J., Wallach J. (1999). Recent trends in the biochemistry of surfactin. Appl. Microbiol. Biotechnol..

[12] Maier R., Soberón-Chávez G. (2000). *Pseudomonas aeruginosa* rhamnolipids: Biosynthesis and potential applications. Appl. Microbiol. Biotechnol..

[13] Claus S., Van Bogaert I.N. (2017). Sophorolipid production by yeasts: A critical review of the literature and suggestions for future research. Appl. Microbiol. Biotechnol..

[14] Tabuchi Y., Nakamura I., Kobayashi T. (1977). Accumulation of the open-ring acid of spiculisporic acid by *Penicillium spiculisporum* in shake culture. J. Ferment. Technol..

[15] Gama Y., Yasumoto M., Suzuki H., Ishigami Y. (1989). Highly selective conversion of spiculisporic acid to ester, amide and imide derivatives. J. Jpn. Oil Chem. Soc..

[16] Ongena M., Jourdan E., Adam A., Paquot M., Brans A., Joris B., Arpigny J.-L., Thonart P. (2007). Surfactin and fengycin lipopeptides of *Bacillus subtilis* as elicitors of induced systemic resistance in plants. Environ. Microbiol..

[17] Sanchez L., Courteaux B., Hubert J., Kauffmann S., Renault J.H., Clément C., Baillieul F., Dorey S. (2012). Rhamnolipids elicit defense responses and induce disease resistance against biotrophic, hemibiotrophic, and necrotrophic pathogens that require different signaling pathways in *Arabidopsis* and highlight a central role for salicylic acid. Plant Physiol..

[18] Mian G., Musetti R., Belfiore N., Boscaro D., Lovat L., Tomasi D. (2023). Chitosan application reduces downy mildew severity on grapevine leaves by positively affecting gene expression pattern. Physiol. Mol. Plant Pathol..

[19] van Loon L.C., Rep M., Pieterse C.M. (2006). Significance of inducible defense-related proteins in infected plants. Annu. Rev. Phytopathol..

[20] Aziz A., Heyraud A., Lambert B. (2004). Oligogalacturonide signal transduction, induction of defense-related responses and protection of grapevine against *Botrytis cinerea*. Planta.

[21] Derckel J.P., Baillieul F., Manteau S., Audran J.C., Haye B., Lambert B., Legendre L. (1999). Differential induction of grapevine defenses by two strains of *Botrytis cinerea*. Phytopathology.

[22] Uknes S., Dincher S., Friedrich L., Negrotto D., Williams S., Thompson-Taylor H., Potter S., Ward E., Ryals J. (1993). Regulation of pathogenesis-related protein-1a gene expression in tobacco. Plant Cell.

[23] Manners J.M., Penninckx I.A., Vermaere K., Kazan K., Brown R.L., Morgan A., Maclean D.J., Curtis M.D., Cammue B.P., Broekaert W.F. (1998). The promoter of the plant defensin gene PDF1.2 from Arabidopsis is systemically activated by fungal pathogens and responds to methyl jasmonate but not to salicylic acid. Plant Mol. Biol..

[24] Vlot A.C., Sales J.H., Lenk M., Bauer K., Brambilla A., Sommer A., Chen Y., Wenig M., Nayem S. (2021). Systemic propagation of immunity in plants. New Phytol..

[25] Otzen D.E. (2017). Biosurfactants and surfactants interacting with membranes and proteins: Same but different?. Biochim. Biophys. Acta Biomembr..

[26] Zipfel C., Robatzek S., Navarro L., Oakeley E.J., Jones J.D., Felix G., Boller T. (2004). Bacterial disease resistance in *Arabidopsis* through flagellin perception. Nature.

[27] Gorin P.A.J., Spencer J.F.T., Tulloch A.P. (1961). Hydroxy fatty acid glycosides of sophorose from *Torulopsis magnoliae*. Can. J. Chem..

[28] Kobayashi Y., Fukuoka T. (2025). Sophorolipids, commercialized glycolipid biosurfactants: Derivatives, component analysis, and applications. J. Am. Oil. Chem. Soc..

[29] De O Caretta T., I Silveira V.A., Andrade G., Macedo F., Celligoi M.A.P.C. (2022). Antimicrobial activity of sophorolipids produced by *Starmerella bombicola* against phytopathogens from cherry tomato. J. Sci. Food Agric..

[30] Hipólito A., Silva R.A.A., Caretta T.O., Silveira V.A.I., Amador I.R., Panagio L.A., Borsato D., Celligoi M.A.P.C. (2020). Evaluation of the antifungal activity of sophorolipids from *Starmerella bombicola* against food spoilage fungi. Biocatal. Agric. Biotechnol..

[31] Al-Mutar D.M.K., Noman M., Abduljaleel Alzawar N.S., Azizullah, Li D., Song F. (2023). Cyclic lipopeptides of *Bacillus amyloliquefaciens* DHA6 are the determinants to suppress watermelon *Fusarium* wilt by direct antifungal activity and host defense modulation. J. Fungi.

[32] Li Y., Héloir M.C., Zhang X., Geissler M., Trouvelot S., Jacquens L., Henkel M., Su X., Fang X., Wang Q. (2019). Surfactin and fengycin contribute to the protection of a *Bacillus subtilis* strain against grape downy mildew by both direct effect and defence stimulation. Mol. Plant Pathol..

[33] Rodrigues A.I., Gudiña E.J., Abrunhosa L., Malheiro A.R., Fernandes R., Teixeira J.A., Rodrigues L.R. (2021). Rhamnolipids inhibit aflatoxins production in *Aspergillus flavus* by causing structural damages in the fungal hyphae and down-regulating the expression of their biosynthetic genes. Int. J. Food Microbiol..

[34] Fungicide Resistance Action Committee CAA Working Group Report. https://www.frac.info/frac-teams/working-groups/caa-fungicides#open-tour.

[35] Lorenzo O., Chico J.M., Sánchez-Serrano J.J., Solano R. (2004). *JASMONATE-INSENSITIVE1* encodes a MYC transcription factor essential to discriminate between different jasmonate-regulated defense responses in *Arabidopsis*. Plant Cell.

[36] Furuya S., Mochizuki M., Saito S., Kobayashi H., Takayanagi T., Suzuki S. (2010). Monitoring of QoI fungicide resistance in *Plasmopara viticola* populations in Japan. Pest Manag. Sci..

[37] Aoki T., Aoki Y., Ishiai S., Otoguro M., Suzuki S. (2017). Impact of *Bacillus cereus* NRKT on grape ripe rot disease through resveratrol synthesis in berry skin. Pest Manag. Sci..

